# Low anterior resection syndrome after rectal resection management: multicentre randomized clinical trial of transanal irrigation with a dedicated device (cone catheter) *versus* conservative bowel management

**DOI:** 10.1093/bjs/znad078

**Published:** 2023-03-28

**Authors:** Guillaume Meurette, Jean-Luc Faucheron, Eddy Cotte, Quentin Denost, Guillaume Portier, Jerôme Loriau, Andreas Wolff Hansen, Eric Vicaut, Zaher Lakkis

**Affiliations:** Department of Visceral Surgery, Hôpitaux Universitaires de Genève, Geneva, Switzerland; Colorectal Surgery Unit, Visceral Surgery and Acute Care Surgery Department, CHU Grenoble Alpes, Grenoble, France; Department of Digestive and Oncological Surgery, Lyon Sud University Hospital, Hospices Civils de Lyon, Lyon, France; Department of Visceral Surgery, Hospital Pierre Bénite, CHU Lyon, Lyon, France; Department of Visceral Surgery, CHU Toulouse, Toulouse, France; Department of Visceral Surgery, Groupe Hospitalier Saint Joseph, Paris, France; Coloplast, Humlebaek, Denmark; Department of Biostatistics Hospital Saint-Louis, Paris, France; Department of Digestive Surgical Oncology, University Hospital of Besançon, Besançon, France

## Introduction

The incidence of low anterior resection syndrome (LARS) has increased over time owing to an increasing rate of sphincter-sparing surgery^1–4^. The standard of care (SOC) for LARS relies on transit control (low-fibre diet); pelvic floor exercises, and medications. Colonic irrigation has recently been evaluated, with promising results^5^. A newly developed Peristeen^®^ cone catheter (Coloplast, Humlebaek, Denmark) has been designed with specific clinical and anatomical considerations in mind for patients with LARS. This anatomical cannula does not have any inflatable balloon; instead, a cone is used that the patient keeps digital contact with during the irrigation. The aim of this randomized trial was to evaluate the superiority of transanal irrigation (TAI) with the cone catheter against traditional SOC.

## Methods

This multicentre (7 centres) open-label RCT included patients with a low colorectal or coloanal anastomosis who had major LARS (LARS score at least 30)^6^ at least 3 months after stoma closure. After rectal examination and assessment of the anastomosis, patients were allocated randomly to either TAI or SOC alone. All centres had previous expertise in Peristeen^®^ balloon catheter use. The primary objective was to demonstrate the superiority of TAI over SOC in improving the LARS score at 3 months. Secondary endpoints were safety of the device and adverse events, and patient satisfaction rates, including daily time spent on bowel management and Faecal Incontinence Quality of Life (FIQL) scale scores.

SOC was delivered by the treating physician to every patient based on a pathway of bowel management, including a low-fibre diet, laxatives and/or loperamide, physiotherapy (biofeedback and pelvic floor retraining), and small-volume enemas (over 150 ml). For patients allocated to TAI, specific education and training was undertaken by a dedicated nurse or the treating physician in a consultation dedicated to patient education. Subsequently, irrigations were administered by the patient daily, starting with a maximum 1-litre enema, with a self-reported diary being used to record daily irrigation efficacy.

A minimal difference in LARS score of 7 between the TAI and SOC groups was deemed clinically relevant. Based on data from a previous study^7^ with a balloon catheter, it was estimated that 13 patients per group (17 patients with a hypothetical 20 per cent drop-out rate) would give sufficient power to establish superiority. The primary endpoint was LARS score analysed by unpaired *t* test. Statistical analysis was performed with SAS^®^ version 9.4 (SAS Institute, Cary, NC, USA).

## Results

**Fig. 1:**
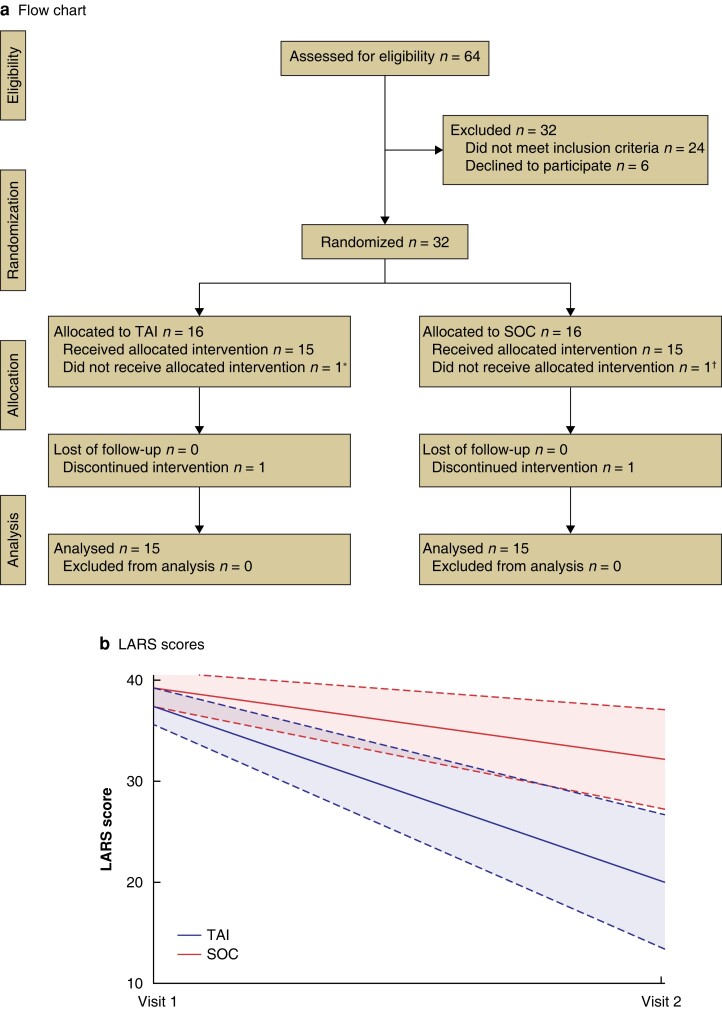
Study flow chart and changes in lower anterior resection syndrome score from baseline

Some 64 patients with severe LARS were considered eligible. Of these, 32 (22 men) met the inclusion criteria and were randomized. One patient in each group was excluded (1 performed TAI in SOC group and was excluded; 1 deferred performing the enemas in TAI group and was no longer eligible) (*Fig. 1a*). Mean age was 63.1 years. Characteristics of the patients are summarized in *Table S1*.

**a** Study flow chart. *Patient delayed management and no longer met the expected time to initiation of irrigation. †Patient initiated irrigation on his own and was excluded from study. **b** Mean(s.d.) changes in lower anterior resection syndrome (LARS) score from baseline. Shaded areas represent 95% confidence intervals. Visit 1, baseline; visit 2, end of 3 months’ follow-up. TAI, transanal irrigation; SOC, standard of care.

By 3 months, the mean LARS score in the overall population had decreased from 38.3 to 26.5 (*P* < 0.001). The comparison between TAI and SOC is summarized in *Table 1*. The mean LARS score was significantly lower in the TAI group at study termination (*P* = 0.008). Because of the small number of patients, a sensitivity analysis was performed (Wilcoxon non-parametric test) that confirmed this result. The change from baseline between the treatments was analysed ad hoc using a linear regression model (*Fig. 1b*); this confirmed the superiority of TAI with cone catheter over SOC. The results were particularly significant for leaks and clustering, but there was an absolute improvement in four of five categories of the LARS score (*Table S2*). The coping behaviour subscale of the FIQL scale showed a difference between groups (*P* = 0.047) (*Table 1*).

**Table 1:** Results for primary and secondary endpoints: comparison between groups

	Assessment time	TAI (*n* = 15)	SOC (*n* = 15)	*P*
**LARS score, mean (95% c.i.)**	Baseline	37.4 (35.5, 39.2)	39.2 (37.5, 40.9)	−
	3 months	21.3 (14.7, 27.8)	32.2 (27.3, 37.1)	0.008*
No. with major LARS	Baseline	15	15	−
	3 months	5	10	0.026†
Visits to toilet per day, mean (95% c.i.)	3-month mean	1.4 (0.9, 2.1)	3.8 (2.7, 5.4)	<0.001
Visits to toilet at night, mean (95% c.i.)	3-month mean	0.29 (0.18, 0.48)	1.14 (0.58, 2.26)	<0.001
Time spent on bowel management and bowel incontinence episodes per day (min), mean (95% c.i.)	3-month mean	32.8 (31.2, 34.4)	68.1 (62.1, 74.2)	<0.001
**FIQL score, mean (95% c.i.)**				
Lifestyle	Baseline	2.19	2.12	−
	3 months	3.1 (2.65, 3.55)	2.6 (2.06, 3.15)	ns
Coping/behaviour	Baseline	1.94	1.81	−
	3 months	2.95 (2.55, 3.35)	2.37 (1.91, 2.82)	0.047
Depression/self-perception	Baseline	3.47	2.79	−
	3 months	3.41 (2.87, 3.95)	3.23 (2.76, 3.71)	ns
Embarrassment	Baseline	2.29	2.16	−
	3 months	2.98 (2.57, 3.38)	2.76 (2.26, 3.27)	ns
**EQ-5D-5L™, mean (95% c.i.)**				
			
Utility score	Baseline	0.88	0.83	−
	3 months	0.92 (0.86, 0.97)	0.88 (0.81, 0.95)	ns
VAS score	Baseline	75.8	67.5	−
	3 months	82.8 (70.2, 95.5)	71.4 (57.8, 85.0)	ns
Wexner score, mean (95% c.i.)	Baseline	17.9	19.2	−
	3 months	12.2 (9.6, 14.8)	15.1 (12.7, 17.5)	ns
Satisfaction score (scale 0–10), mean (95% c.i.)	Baseline	6.6	5.9	−
	3 months	8.6 (7.4, 9.7)	6.7 (5.1, 8.3)	0.048

TAI, transanal irrigation; SOC, standard of care; LARS, lower anterior resection syndrome; FIQL, Faecal Incontinence Quality of Life; EQ-5D-5L™, EuroQol Five Dimensions 5L (EuroQol Group, Rotterdam, the Netherlands); VAS, visual analogue scale. *Unpaired *t* test, except †? test.

A total of 17 adverse events occurred in 14 patients (none severe). Seven events in five patients were related to irrigation pain at the anus (2), and abdominal spasms and transit disturbance during irrigation (5). The mean(s.d.) volume of water used per irrigation per day was 718(135) ml. The time spent on bowel management per day decreased significantly in TAI group as compared to SOC group (*Figs S1* and *S2*). During the 3 months, only nine use events were reported: water leakage (2), immediate reuse of cone (3), and low water flow/pressure (4). All patients who used a Peristeen^®^ cone requested to continue with this treatment at the end of the study.

## Discussion

In this study, there was a marked improvement in LARS score in patients using TAI compared with the SOC group at study termination after 3 months. These positive results of TAI with a cone mirror outcomes reported in other preliminary studies with a balloon catheter^5,7–9^.

Selection of the best candidates for colonic irrigation is still a matter of debate, and the optimal postoperative period for starting the irrigations has not been clearly defined. In the present study, the majority of patients had chronic LARS, and so future studies should validate earlier use of the device. Education when introducing TAI is crucial, and the use of a cone catheter requires training for patients to orientate the device and maintain the catheter during irrigation. The major advantage is the safety of the anatomical design. Other studies with a balloon catheter have reported greater difficulties with the use of TAI. Pieniowski *et al*.^9^ reported a rate of difficulties of 20 per cent for catheter insertion or irrigation even after 12 months. Therefore, using a dedicated anatomical device could improve the ease of use and potentially decrease the drop-out rate. Additionally, all patients allocated to the Peristeen^®^ cone group decided to continue treatment after study termination. This was possibly related to the improvement in symptoms and simplicity of the procedure, with fewer user steps as there was no balloon to inflate.

The risk of rectal perforation during TAI should be acknowledged. Perforations were reported when TAI was used for other indications, including neurogenic sources of incontinence^10,11^. An audit of the risk of perforation during TAI estimated an average risk of 1 in 167 000 for bowel perforation. This risk could be higher in patients who had undergone pelvic radiotherapy and/or rectal surgery previously, and particularly with a pouch or side-to-end anastomosis^12^. The Peristeen^®^ cone catheter is therefore suggested as a better option for patients with a history of rectal surgery owing to the anatomical design.

Despite these promising results, the present study is limited by the short follow-up of 3 months. These results must be confirmed with longer follow-up to allow conclusions to be drawn regarding the long-term efficacy of TAI and evaluation of the patient’s comfort with the cone^9^. Moreover, the wide range of duration of symptoms could have been a source of bias, and spontaneous evolution influenced the response to management as mentioned by Varghese *et al*.^13^. It is generally accepted that LARS symptoms can improve spontaneously over time and then perception of severity could also be modified^13^. Nevertheless, studies have also reported adverse symptoms and severe impairment of quality of life for as long as 15 years^3^. The patients enrolled in the present study were all naive to bowel management programmes or colonic irrigations. This probably increased the homogeneity between groups. Finally, the LARS score is a validated, but probably not ideal, tool for assessment of the LARS in all patients; future research, including qualitative assessment and patient-reported outcome, are mandatory to overcome bias owing to the long follow-up period and its impact on the LARS score.

## Supplementary Material

znad078_Supplementary_Data

## Data Availability

All the data reported in this study are available for further information (clinical trial identifier NCT04586634).
