# Supplementary material for: Low anterior resection syndrome after rectal resection management: multicentre randomized clinical trial of transanal irrigation with a dedicated device (cone catheter) versus conservative bowel management
Source: Br J Surg. 2023 Mar 28;110(9):1092–5. doi: 10.1093/bjs/znad078 (PMC10416684; doi:10.1093/bjs/znad078)
Supplement: znad078_Supplementary_Data [file znad078_supplementary_data.zip › Supplementary_Material.docx]

**Low anterior resection syndrome after rectal resection management: a multicenter randomized, controlled clinical trial of transanal irrigation with a dedicated device (Cone catheter) versus conservative bowel management**

Guillaume Meurette^1^, Jean-Luc Faucheron^2^, Eddy Cotte^3^, Quentin Denost^4^, Guillaume Portier^5^, Jerôme Loriau^6^, Andreas Wolff Hansen^7^, Eric Vicaut^8^, Zaher Lakkis^9^

1 Department of digestive surgery, CHU Nantes, Hôtel-Dieu, France

2 Colorectal Surgery Unit, Visceral Surgery and Acute Care Surgery Department, CHU Grenoble Alpes, Grenoble, France

3 Department of Digestive and Oncological Surgery, Lyon Sud University Hospital, Hospices Civils de Lyon, Lyon, France

4 Department of visceral surgery, Hospital Pierre Bénite, CHU Lyon, France

5 Department of visceral surgery, CHU Toulouse, France

6 Department of visceral surgery, Groupe hospitalier Saint Joseph, Paris, France

7 Coloplast A/S, Humlebaek, Denmark,

8 Department of biostatistics Hospital Saint-Louis, Paris

9 Department of Digestive Surgical Oncology, University Hospital of Besançon, Besançon, France

Corresponding author: [guillaume.meurette@chu-nantes.fr](mailto:guillaume.meurette@chu-nantes.fr)

**Supplementary Materials – Index:**

| **Supplementary Figures and Tables** |  |
| --- | --- |
| Table S1 (page2)  Table S2 (page 3) |  |
| Figure S1 (page 4)  Figure S2 (page 5) |  |
|  |  |
|  |  |
|  |  |
|  |  |
|  |  |
|  |  |
|  |  |
|  |  |
|  |  |
|  |  |
|  |  |

**Table S1**: Baseline characteristics of study population.

|  |  | **Peristeen Cone** (n=15) | **SOC** (n=15) | **Total** (n=30) |
| --- | --- | --- | --- | --- |
| **Gender** | Women, n (%) | 2 (13.3) | 6 (40.0) | 8 (26.7) |
|  | Men, n (%) | 13 (86.7) | 9 (60.0) | 22 (73.3) |
| **Age (year)** | Mean (SD) | 63.3 (12.9) | 62.9 (10.1) | 63.1 (11.4) |
|  | Min; Max | 37 ; 81 | 41 ; 73 | 37 ; 81 |
|  | Median | 67.0 | 66.0 | 66.5 |
| **Type of surgery** | Total mesorectal excision, n (%) | 15 (100.0) | 15 (100.0) | 30 (100.0) |
|  | Partial mesorectal excision, n (%) | 0 (0.0) | 0 (0.0) | 0 (0.0) |
| **Preoperative radiation** | No, n (%) | 4 (26.7) | 3 (19.4) | 7 (23.3) |
|  | Yes, n (%) | 11 (73.3) | 12 (80.6) | 23 (76.7) |
| **Reconstruction type** | Pouch, n (%) | 6 (40.0) | 6 (40.0) | 12 (40.0) |
|  | Straight anastomosis, n (%) | 9 (60.0) | 9 (60.0) | 18 (60.0) |
| **T stage** | T0, n (%) | 3 (20.0) | 0 (0.0) | 3 (10.0) |
|  | T1, n (%) | 6 (40.0) | 3 (20.0) | 9 (30.0) |
|  | T2, n (%) | 3 (20.0) | 7 (46.7) | 10 (33.3) |
|  | T3, n (%) | 3 (20.0) | 4 (26.7) | 7 (23.3) |
| **Duration of symptoms (months)** | Mean (SD) | 18.4 (13.3) | 36.4 (38.0) | 27.4 (29.4) |
|  | Min; Max | 3 ; 48 | 3 ; 156 | 3 ; 156 |
|  | Median | 16.0 | 29.0 | 22.0 |
| **Current bowel management** | Abdominal massage, Yes n (%) | 0 (0.0) | 1 (6.7) | 1 (3.3) |
|  | Dietary modification/restriction, Yes n (%) | 5 (33.3) | 9 (60.0) | 14 (46.7) |
|  | Treatment with loperamide, Yes n (%) | 10 (66.7) | 9 (60.0) | 19 (63.3) |
|  | Suppositories or small enemas, Yes n (%) | 0 (0.0) | 1 (6.7) | 1 (3.3) |
|  | Manual digital evacuation/stimulation, Yes n (%) | 0 (0.0) | 1 (6.7) | 1 (3.3) |
|  | No specific treatment, Yes n (%) | 1 (6.7) | 1 (6.7) | 2 (6.7) |
|  | Other, Yes n (%) | 8 (53.3) | 7 (40.0) | 14 (46.7) |
|  | Medication/Dietary supplements*, n (%) | 7 (46.6) | 4 (26.6) | 11 (36.6) |
|  | Biofeedback/ perineal rehabilitation, n (%) | 2 (6.7) | 5 (33.3) | 7 (46.6) |

**Table S2**: Comparison of LARS subscales between TAI and SOC groups

|  | **Baseline** | | **Follow-up** | |  |
| --- | --- | --- | --- | --- | --- |
|  | SOC | TAI | SOC | TAI | P-value |
| LARS SCORE DOMAIN |  |  |  |  | - |
| Flatus (0,4,7) | 6.8 (0.8) | 6.4 (1.3) | 6.1 (2.1) | 4.7 (3.1) | P = 0.19 |
| Leakage (0,3,3) | 3.0 (0.0) | 2.8 (0.8) | 2.8 (0.8) | 1.6 (1.5) | P = 0.02 |
| Frequency  (4,2,0,5) | 2.7 (2.0) | 2.4 (1.4) | 1.9 (1.6) | 1.9 (2.3) | P = 1.00 |
| Clustering (0,9,11) | 11.0 (0.0) | 10.9 (0.5) | 9.9 (2.9) | 6.5 (4.9) | P = 0.03 |
| Strong urge (0,11,16) | 15.7 (1.3) | 14.9 (2.1) | 11.5 (6.6) | 6.5 (7.4) | P = 0.06 |

Mean (SD)

**
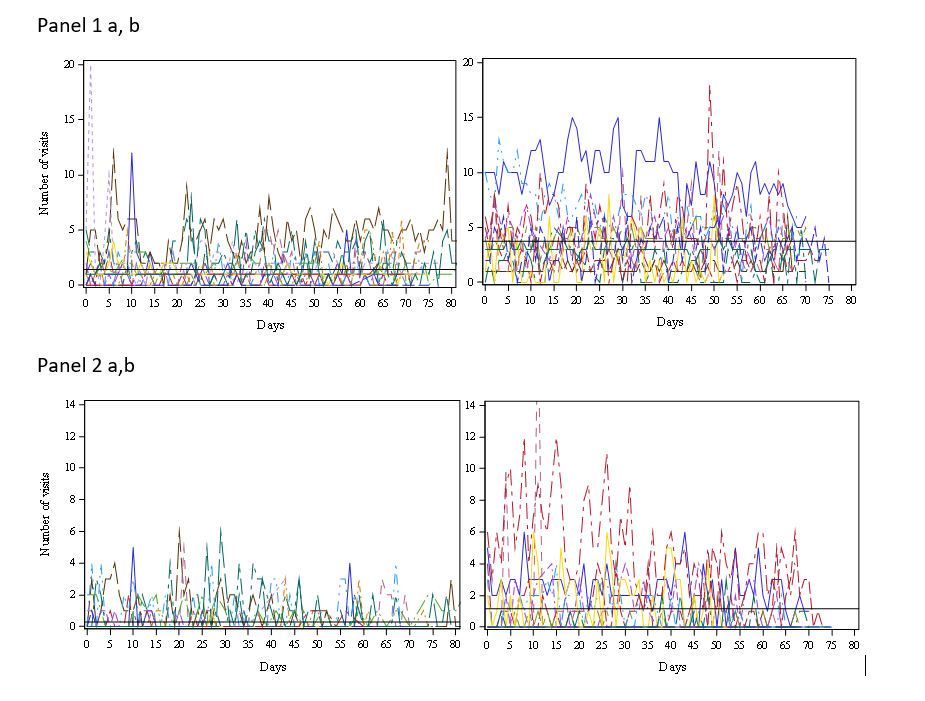
**

**Fig S1**: Number of visits to toilet for defecation by individual patients into (a) Peristeen cone group and (b) SOC group; during the day (Panel 1); during the night (Panel 2). The horizontal line represent the mean number of visits per day

**
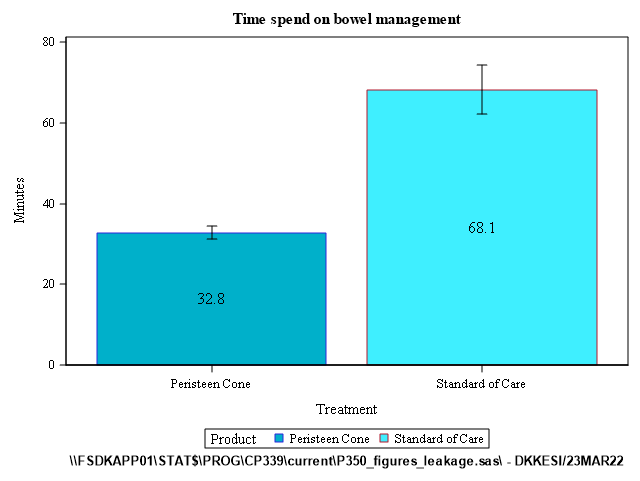
**

**Fig S2**: Mean time spent on bowel management and bowel incontinence episodes by groups (p<0.0001)
